# Functional Class II Treatment Simultaneous With Selective Reduction of Talon Cusps: A Case Report

**DOI:** 10.1002/ccr3.70015

**Published:** 2024-12-15

**Authors:** Sahar Yaghoutiazar, Atiye Yadegari, Saharnaz Esmaeili, Alireza Hajizadeh, Soheil Shahbazi

**Affiliations:** ^1^ Department of Pediatric Dentistry, Faculty of Dentistry Hamadan University of Medical Sciences Hamadan Iran; ^2^ Dentofacial Deformities Research Center, Research Institute of Dental Sciences Shahid Beheshti University of Medical Sciences Tehran Iran; ^3^ Department of Endodontics, Faculty of Dentistry Hamadan University of Medical Science Hamadan Iran; ^4^ Dental Research Center Shahid Beheshti University of Medical Sciences Tehran Iran

## Abstract

Selective reduction of bilateral nonsyndromic talon cusps in an 11‐year‐old class II patient enabled successful mandibular repositioning with a twin block appliance. This approach achieved desired orthodontic outcomes without causing pain or sensitivity following reduction, highlighting a novel strategy for managing talon cusps in orthodontic treatment.

## 1 Introduction

Talon cusp (TC) is characterized as a developmental dental anomaly that manifests as a cusp‐like projection extending from the cingulum to the incisal edge of primary or permanent anterior teeth [1]. Although the exact etiology behind TC remains contentious, the equilibrium between environmental and genetic predispositions has been highlighted as a potential cause [2]. Some studies also suggest that hyperactivity of the enamel organ during tooth morphodifferentiation could be a contributing factor [3]. According to the current literature, the global prevalence of TC is estimated to be 1.67%, with no significant gender predilection [1].

Mandibular deficiency is one of the most common facial growth disorders, serving as the leading etiology of class II profile [4]. Several approaches are available for correcting mandibular deficiency, including functional appliances, fixed braces, growth modification devices, orthognathic surgeries, and distraction osteogenesis [5]. Several activator devices and functional appliances have been introduced in orthodontic treatments, including the twin block, Seifi appliance, Herbst appliance, and face mask, among others [6, 7]. The twin block appliance is commonly employed to correct mandibular deficiency, yielding promising outcomes in preadolescent children [5]. The functional principle of twin block involves directing the mandible forward upon mouth closure, thereby stimulating condylar growth in an upward and backward direction [8].

Following mandibular advancement, a talon cusp may interfere with the new anterior position of the mandibular teeth, potentially causing a more posterior positioning of the mandible upon closure. In the long term, this can result in a relapse of mandibular advancement achieved through twin block appliance therapy. The current study aimed to report the successful management of a patient with bilateral non‐syndromic TCs on permanent maxillary lateral incisors simultaneous with twin block therapy.

## 2 Case History/Examination

An 11‐year‐old male patient visited the Department of Orthodontics, Hamadan University of Medical Sciences, Hamadan, Iran, with the primary concern of having his teeth aligned. The child's medical background revealed no notable issues. No incidents of dental trauma were reported during the child's developmental period, and no dental anomalies other than TC were observed. An extensive general examination was undertaken to eliminate the possibility of any syndromic connections or underlying health conditions that could be related to the dental observations.

**FIGURE 1:**
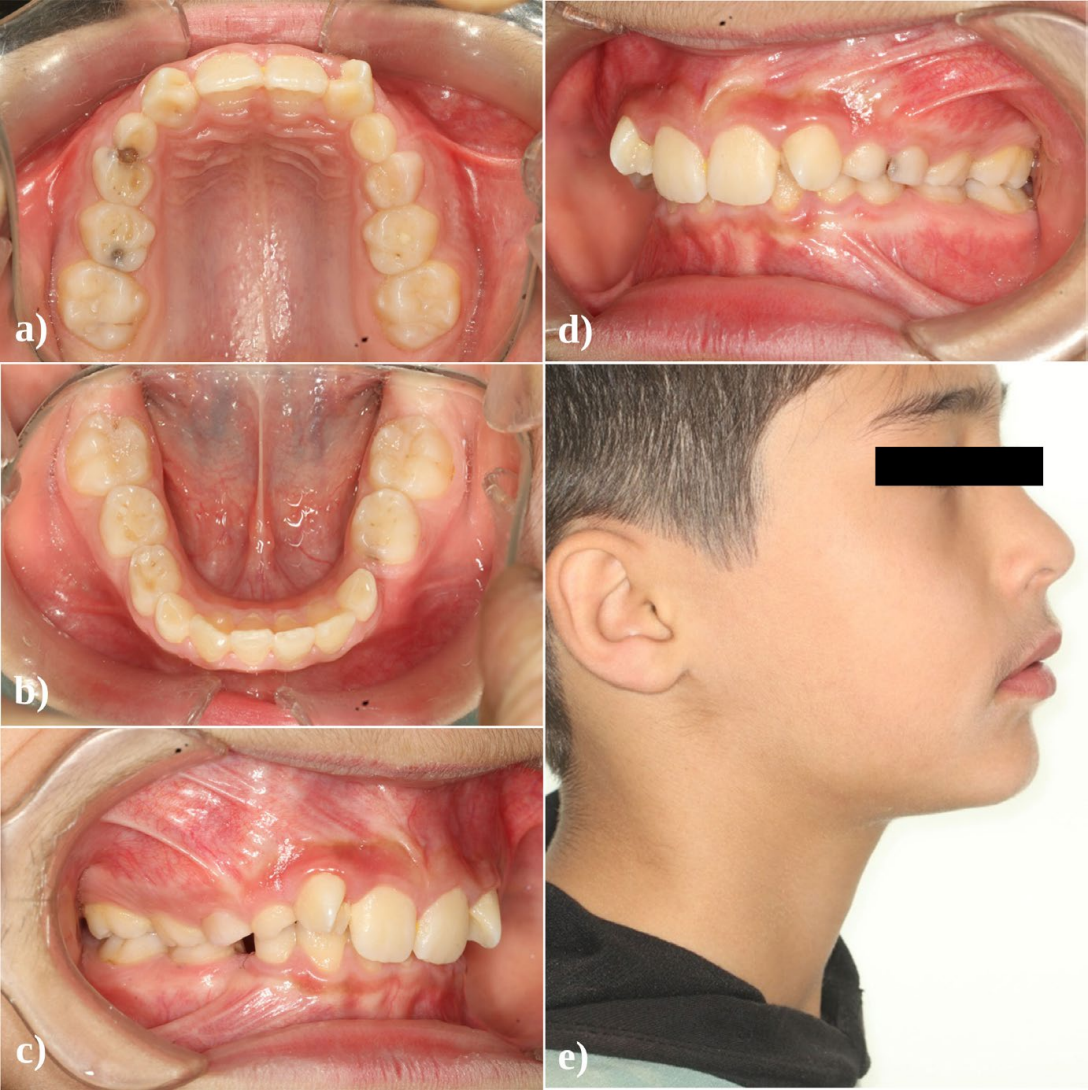
(a–d) Pretreatment intraoral photographs. (e) Pretreatment profile view.

The patient was at the mixed dentition stage and had a healthy periodontal condition. Arrested caries were observed on the left primary maxillary canine and primary molars as well as on the right primary mandibular second molar. Both permanent maxillary lateral incisors exhibited a well‐delineated, anomalous cusp‐like projection on the palatal side, closely resembling the appearance of a “true talon” (Figure 1). Electric and thermal pulp testing confirmed the vitality of these two teeth.

**FIGURE 2:**
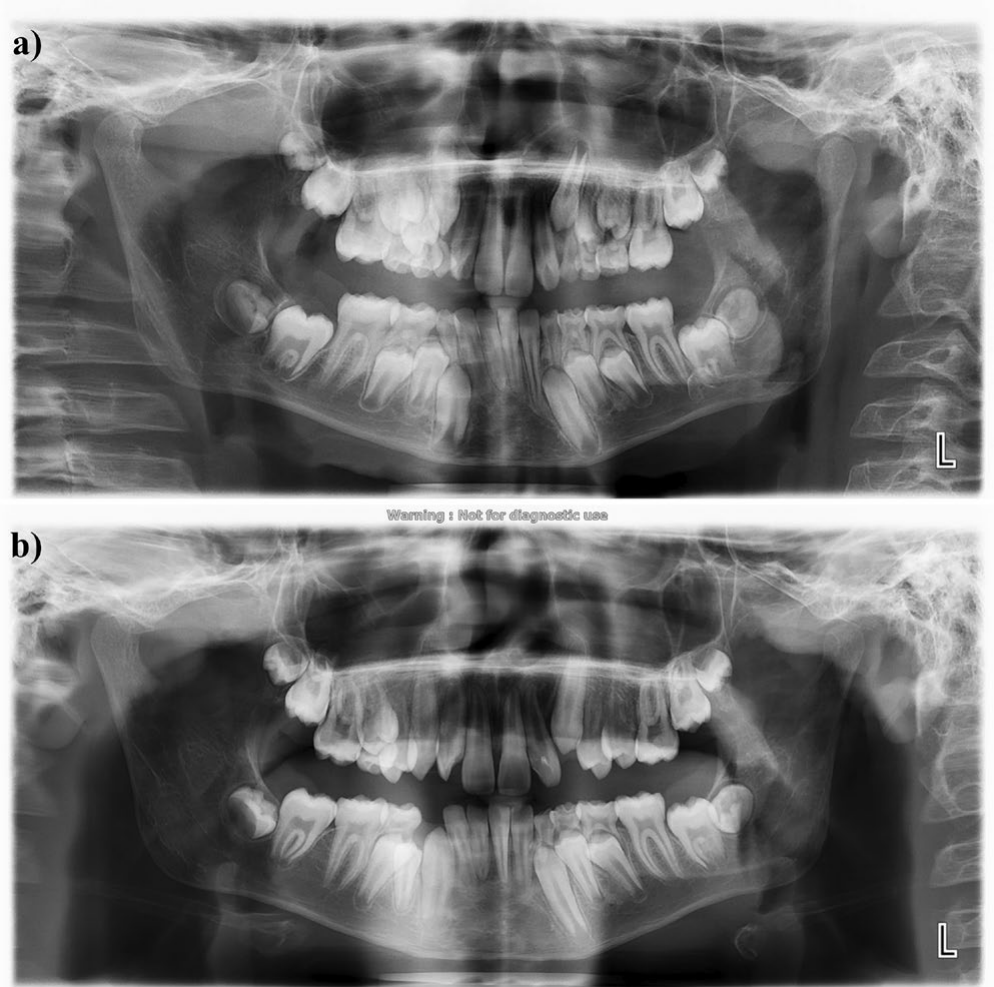
(a) Pretreatment panoramic view. (b) Posttreatment panoramic view.

**FIGURE 3:**
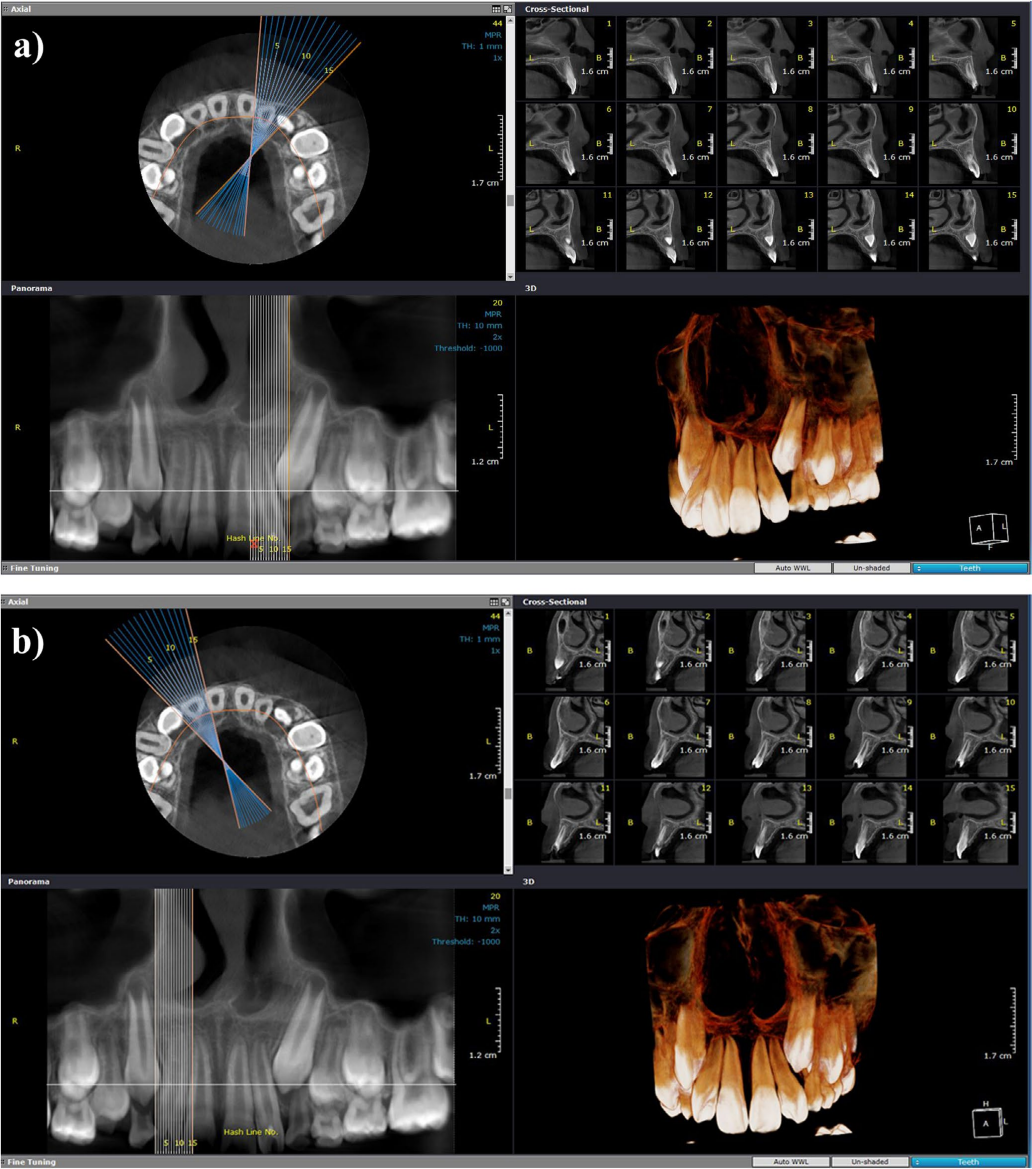
(a, b) Pretreatment computed cone‐beam tomography (CBCT).

Radiographic assessments revealed a V‐shaped radiopacity on the crowns of the right and left maxillary lateral incisors, with the apex pointing toward the incisal edges (Figures 2 and 3). The apices of both maxillary lateral incisors were found to be open. The convergence of clinical and radiographic findings led to the definitive diagnosis of non‐syndromic TC.

Facial examination delineated symmetrical features, a convex profile, a 2 mm gingival display upon smiling, and 1 mm visibility of the maxillary incisors in the resting state. Lip alignment was found to be satisfactory with normal mentalis muscle tonicity.

Space analysis during the intraoral assessment revealed a pronounced deficiency in the mandible, further complicated by the rotation of the maxillary lateral incisors, which also featured TCs. While dental crowding was evident within both arches, the difference in tooth size arch length discrepancy was −2.25 mm in the mandible and − 2.5 in the maxilla.

**FIGURE 4:**
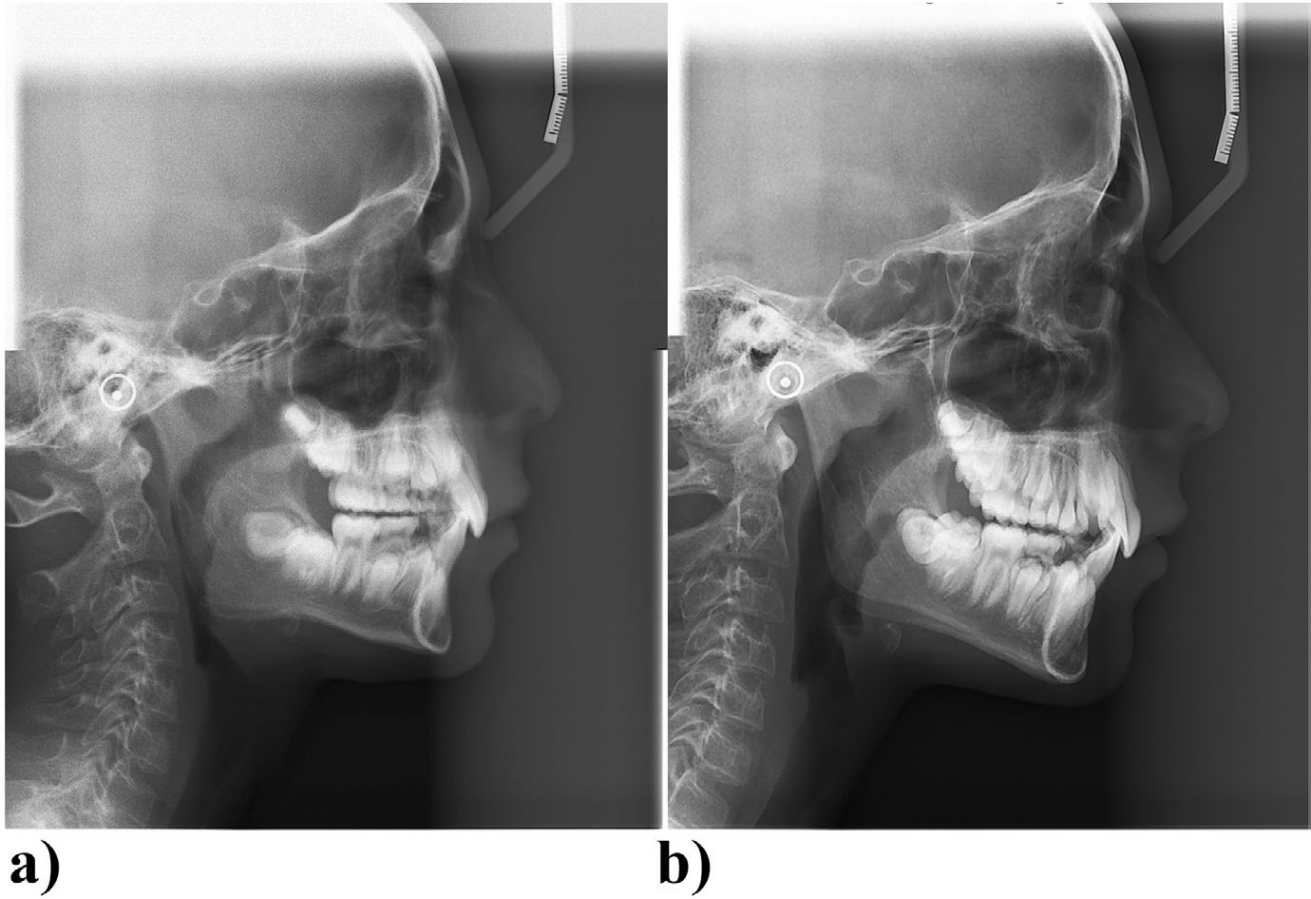
(a) Pretreatment lateral cephalogram. (b) Posttreatment lateral cephalogram.

The developmental examination revealed a more complex picture, highlighting mandibular deficiency that aligned with a skeletal class II division 2 presentation and bilateral class II molar relationships. Based on Steiner cephalometric analysis, the growth pattern was horizontal, contributing to a skeletal deep bite. The maxillary incisors were noted for their retroclination, and there was a marked protrusion of the mandibular lip toward the E line as well as increased overjet (Figures 1 and 4). The initial measurements of overbite and overjet were 4 mm and 3 mm, respectively.

## 3 Methods

A principal component of the orthodontic strategy involved the twin block appliance, which was selected for its proficiency in facilitating mandibular advancement. The patient was instructed to wear the appliance 24 h a day, except while eating. To address the potential interference of talon cusps with the anticipated advanced position of the mandibular teeth, a scheduled reduction of TCs was performed at 6–8‐week intervals, inducing the production of reactionary dentin. With no local anesthetic applied, a football‐shaped diamond bur was employed in a high‐speed handpiece with water spray to perform the reduction in line with the long axis of the teeth. Furthermore, the functional appliance was also adjusted after each reduction session to adapt to the modified circumstances. At the end of each session, fluoride varnish (Preventa, Aria Dent, Tehran, Iran) was applied to the area, and desensitizing toothpaste was prescribed to protect the enamel and enhance the resistance of the tooth to decay.

Following cone‐beam computed tomography (CBCT) and detection of pulp horns within the TC on the maxillary right lateral incisor, the depth of reduction was confined to 0.5–1 mm, while the TC on the left lateral incisor was devoid of pulpal tissues. As a consequence, the possibility of complete TC reduction was precluded, and a cautious approach was necessary to avoid sensitivity or pulpal exposure.

**FIGURE 5:**
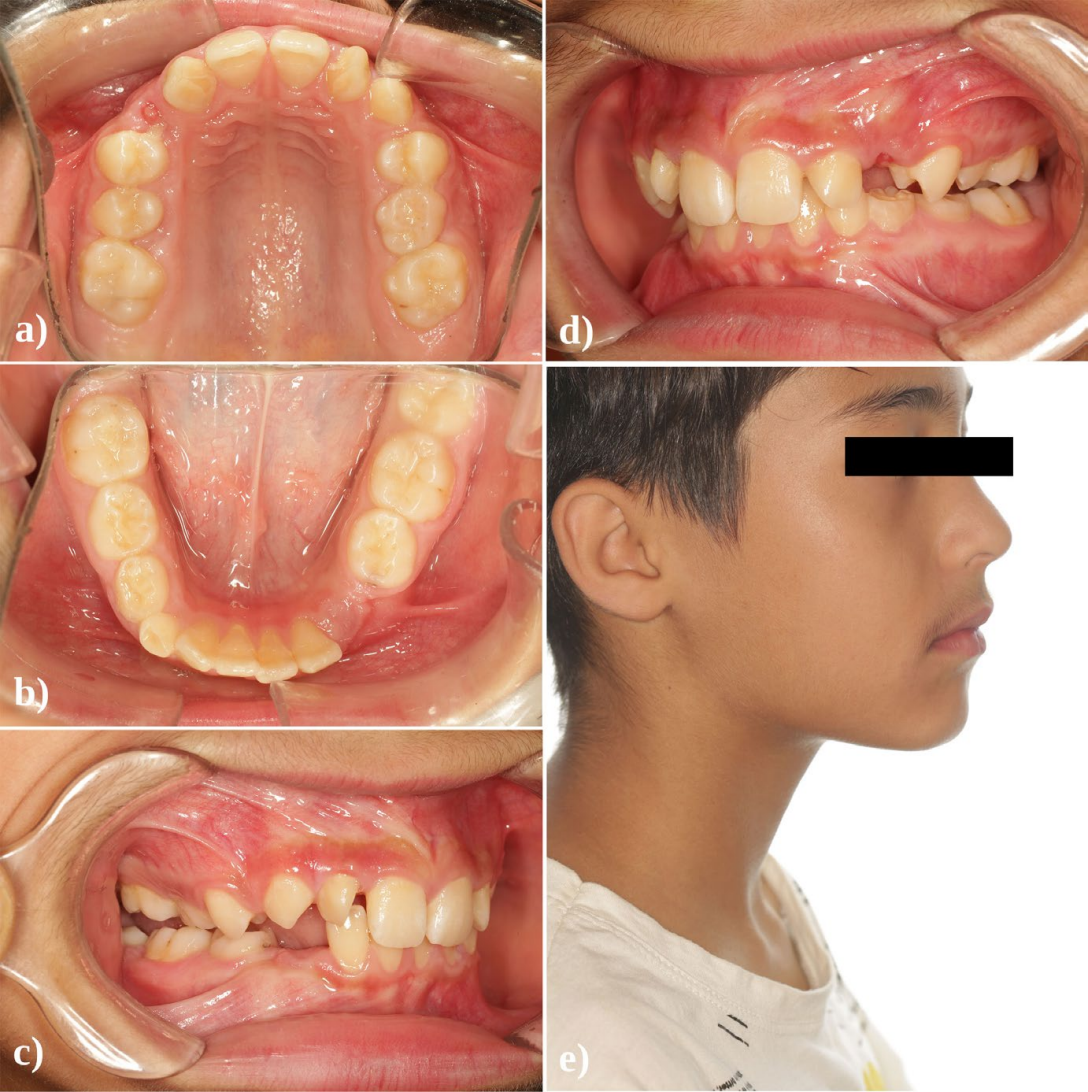
(a–d) Posttreatment intraoral photographs. (e) Posttreatment profile view.

Over a 16‐month period, with steadfast adherence to the treatment protocol involving the twin block appliance and periodic TC reduction, we successfully resolved the mechanical interference and achieved the therapeutic goal of mandibular advancement. Finally, a recession occurred within the pulpal tissues of maxillary right lateral incisor, and no sensitivity or pain was reported by the patient. The posttreatment photographs affirmed the efficacy of the treatment in overcoming the orthodontic challenges presented in this case (Figure 5). The patient was referred to the Department of Orthodontics to proceed with fixed orthodontic treatment.

## 4 Discussion

TC is defined as a dental anomaly presenting as a projection that extends from the cingulum to the incisal edge of anterior teeth [1]. In general, dental anomalies are more prevalent among patients with cleft lip and palate [9], and their distribution varies across different populations. The prevalence in the Caucasian population is documented to be 20.9%, whereas it escalates to 37.8% in Saudi Arabia [10]. Regarding the Iranian population, one study indicated a prevalence of 60.7% for dental anomalies [11], and another study identified that 71.36% of these anomalies pertain to tooth morphology [12]. Consequently, dental practitioners are likely to encounter a range of dental anomalies throughout their careers. This underscores the importance of augmenting their proficiency in diagnosing and managing a diverse array of dental anomalies.

Early intervention with functional appliances for class II subjects can be effective in addressing functional or muscular imbalances, potentially reducing the need for more complex treatments in later years. However, in cases of true mandibular growth deficiency, surgical intervention may still be necessary. Furthermore, synchronization with growth potential is crucial in pediatric patients undergoing orthodontic treatment, as individuals with class II malocclusions typically exhibit shorter adolescent growth spurts [13, 14, 15]. Among the various options available for the presented case, we opted for twin block. This functional appliance serves as a simple tool for children with mandibular deficiency that allows patients to eat and speak comfortably while maintaining acceptable esthetics [16]. The twin block appliance, similar to most other functional appliances, not only induces skeletal changes but also inevitably leads to dental modifications. Retroclination and extrusion of maxillary incisors concomitant with proclination and intrusion of mandibular incisors are among the mentioned changes. Moreover, maxillary molars tend to become distalized and extruded, while mandibular molars shift mesially and extrude [17]. These changes were favorable for the reported case, leading to decreased overjet and overbite. The literature presents a controversy regarding the headgear effect of the twin block appliance [18]; while our findings support the existence of this effect, some studies dispute the presence of the headgear effect associated with twin block therapy.

The presented case was instructed to use the twin block device 24 h a day, except while eating. This protocol represents the most prescribed wear regimen among orthodontists in the United Kingdom [19]. Baheti, Bhad, and Chavan [20] reported that full‐time use of the device can lead to greater skeletal effects, while the dentoalveolar changes do not differ significantly from those observed with part‐time wear. Additionally, full‐time wear of the device results in a more pronounced reduction in the ANB angle and overjet. In contrast, Parekh et al. [21] found no significant differences in skeletal or dental changes between full‐time and part‐time groups, and they recommended part‐time wear for its greater convenience for patients. However, it is important to note that the average duration of wear for full‐time users in Parekh et al.'s study was 12.38 h, while in Baheti et al.'s study, it was 20.86 h, which aligns more closely with our protocol. Clinicians must also consider patient noncompliance, as patients may wear the device for significantly less time than instructed [22]. The presented case was checked every 6–8 weeks to receive TC reduction. It has been shown that, during treatment with the twin block appliance, a shorter checkup interval does not enhance treatment outcomes or increase wear‐time compared to a 6‐week checkup interval [22].

TC potentially acts as a fulcrum and causes a multitude of complications, including esthetic concerns, occlusal interferences, soft tissue irritation, speech difficulties, and tooth mobility [23]. For the present case, selective reduction was performed to eliminate the interference between TCs on maxillary lateral incisors, and forward repositioning of the mandible guided by a twin block. The gradual reduction of TCs over a long period of time stimulates the deposition of reactionary dentin, which protects the pulp horns from exposure and preserves pulp vitality. A 6–8‐week interval has been shown to be adequate for desirable dentin formation [24, 25]. In addition, fluoride varnish was applied at the end of each reduction session to minimize the sensitivity and discomfort experienced by the patient.

Recently, innovative techniques have been introduced that combine the benefits of clear aligners and functional appliances. For instance, precision wings are bilateral inclined planes integrated into Invisalign trays to guide the mandible into a more advanced position, similar to the twin block mechanism, thereby addressing both malocclusion and crowding simultaneously [26, 27]. Perrotti et al. [28] employed the Nuvola OP System to treat transverse maxillary deficiency. This system integrates specific aligners with a device called Freedom, which is designed to be bitten while wearing the aligners. This approach not only facilitates traditional orthodontic movements but also promotes cranial suture adaptation through masticatory muscle contractions. In a separate study by Tepedino et al. [29], the same system was utilized to address open bite cases. Moreover, Segnini et al. [30] introduced the En‐Nova concept, which involves the use of a functional orthodontic appliance worn over the aligners.

While panoramic and lateral cephalometric radiographs are standard two‐dimensional imaging techniques frequently used in orthodontics, three‐dimensional imaging, particularly CBCT, offers enhanced diagnostic accuracy, improved clinical decision‐making, and comprehensive information. Hence, CBCT was employed in the present case to meticulously evaluate the presence and extent of pulp horns within the TCs, thereby preventing inadvertent pulp exposure during the reduction process.

It is essential to acknowledge that the intervention utilized in this case report may not universally suit all patients with differing clinical conditions. For instance, patients who exhibit significant occlusal interference may require a complete reduction of the TC followed by proper endodontic therapy.

## 5 Conclusion

Twin block appliance is commonly used for class II patients with mandibular deficiency. TC is a dental anomaly that may prevent the clinician from repositioning the mandible forward due to the occlusal interference it can create with the mandibular teeth, ultimately leading to a relapse in orthodontic outcomes. Based on our findings, selective reduction of TC during 6–8‐week intervals appears to be an effective strategy for mitigating the interference with anteriorly repositioned mandibular teeth following twin block appliance therapy. In addition, gradual reduction of the tooth structure effectively minimized the patient's sensitivity and pain, thereby eliminating the need for endodontic treatment.

## Author Contributions

**Sahar Yaghoutiazar:** conceptualization, data curation, investigation, methodology. **Atiye Yadegari:** conceptualization, data curation, investigation, methodology. **Saharnaz Esmaeili:** project administration, supervision, writing – original draft. **Alireza Hajizadeh:** conceptualization, investigation. **Soheil Shahbazi:** project administration, supervision, writing – review and editing.

## Ethics Statement

The authors have nothing to report.

## Consent

Written informed consent regarding participation and publication of the findings was acquired from the patient's caregiver.

## Conflicts of Interest

The authors declare no conflicts of interest.

## Data Availability

The datasets used and/or analyzed during the current study available from the corresponding author on reasonable request.
